# Exposure to Armed Conflict in Childhood vs Older Ages and Subsequent Onset of Major Depressive Disorder

**DOI:** 10.1001/jamanetworkopen.2020.19848

**Published:** 2020-11-13

**Authors:** Corina Benjet, William G. Axinn, Sabrina Hermosilla, Paul Schulz, Faith Cole, Laura Sampson, Dirgha Ghimire

**Affiliations:** 1 Department of Epidemiology and Psychosocial Research, National Institute of Psychiatry Ramón de la Fuente Muñiz, Mexico City, Mexico; 2 Population Studies Center, Institute for Social Research, University of Michigan, Ann Arbor; 3 Department of Epidemiology, Boston University School of Public Health, Boston, Massachusetts; 4 Institute for Social and Environmental Research Nepal, Chitwan, Nepal

## Abstract

**Importance:**

This study offers a rare opportunity to evaluate life-course differences in the likelihood of developing major depressive disorder (MDD) after exposure to georeferenced neighborhood-level violence during an armed conflict.

**Objective:**

To examine age cohort (age <11 vs ≥11 years) differences in associations of neighborhood-level violence with subsequent depression onset, independently of individual exposure and other key characteristics.

**Design, Setting, and Participants:**

The Chitwan Valley Family Study is a population-representative panel study (1995 to present) conducted in Western Chitwan in Nepal, a low-income country that experienced a medium-intensity armed conflict from 2000 to 2006. Data for violent events were collected during the armed conflict and were linked to lifetime histories of MDD (collected in 2016-2018). The present cohort study analyzes 10 623 participants within 151 neighborhoods, systematically selected and representative of Western Chitwan. All residents aged 15 to 59 years at MDD assessment were eligible (response rate, 93%). Data analysis was performed from May 2019 to July 2020.

**Exposures:**

Georeferenced number of armed conflict–related physical beatings within 1 km of residential neighborhood.

**Main Outcomes and Measures:**

The main outcome was onset of MDD, as defined by *Diagnostic and Statistical Manual of Mental Disorders* (Fourth Edition), during or after the conflict, stratified by children (aged <11 years) and older individuals (aged ≥11 years), assessed by the Nepal-specific World Mental Health–Composite International Diagnostic Instrument 3.0 with a life history calendar.

**Results:**

In total, 10 623 participants (5745 female [54.08%]; 4074 [38.35%] aged <11 years at the conflict start) contributed 171 899 person-years of exposure to the risk of MDD. Two or more beatings occurred within 1 km of 15 neighborhoods (9.9%). Discrete-time survival models showed that children (but not older individuals) living in neighborhoods with 2 or more beatings had a higher likelihood of developing MDD than those who lived in a community with no beatings (odds ratio, 1.82; 95% CI, 1.17-2.84; *P* = .008); there was also a significant interaction between age group and neighborhood beatings (odds ratio, 1.85; 95% CI, 1.27-2.70; *P* = .001). A confirmatory, multivariable, multilevel matching analysis showed a neighborhood association for children (*z* = −2.66; *P* = .008), but not older individuals (*z* = −0.454; *P* = .65). The mean (SE) incidence of MDD among children living in neighborhoods with 2 or more beatings nearby was 12.69% (2.37%) vs 5.08% (1.56%) in the matched unexposed sample.

**Conclusions and Relevance:**

The youngest individuals may be the most at risk during times of violence, with mental health consequences lasting long after conflicts have subsided and should be a priority for population-level interventions. Future research should consider other disorders, other types of violence, and elderly individuals.

## Key Points

**Question:**

Is neighborhood-level violence associated with incidence of major depressive disorder (MDD) during and after armed conflict?

**Findings:**

In a cohort study including 10 623 participants within 151 neighborhoods in Nepal, the occurrence of 2 or more beatings within 1 km was associated with incidence of MDD in those who were children at the start of the armed conflict (MDD incidence, 12.69% vs 5.08% in a matched unexposed sample), but not for older individuals.

**Meaning:**

Because the youngest children may be the most at risk during times of violence, with mental health consequences lasting long after conflict has subsided, they should be prioritized for population-level interventions.

## Teaser

This cohort study examines the association of age and exposure to neighborhood-level violence with subsequent depression onset in an area with medium-intensity armed conflict.

## Introduction

Although important advances have been made in our understanding of the underpinnings of major depressive disorder (MDD) at the individual level (eg, genes, temperament, and direct experiences of traumatic events), less is understood about wider contextual risk factors, such as those at the neighborhood level.^1^ A large body of research has demonstrated that direct exposure to community violence (as either a victim or a witness) is associated with depressive symptoms and MDD,^2,3^ but fewer studies have evaluated the potential risks of living in a more violent neighborhood, independently of personal exposure to violence.^4,5^ Living in a neighborhood with greater levels of violence may be associated with the development of depression through Beck’s proposed triad of cognitions,^6^ specifically perceiving the world as dangerous or without meaning, the self as worthless, and the future as hopeless. Other mechanisms might include greater daily stress, disruption of social networks, loss of social capital,^7,8^ and in the case of children, the impact of neighborhood violence on caregivers.^9,10^

Many of the existing studies of neighborhood-level violence and depression have used subjective measures of neighborhood violence, such as perceptions of neighborhood safety^11,12,13,14^ or aggregate measures of individual violence reports.^4^ Only a handful of studies have used objective measures, such as official government crime statistics,^5,15,16,17^ and none of those is able to estimate subsequent MDD over the life course.

There have been studies^18,19^ of neighborhood violence and depression in children and a separate, but related, body of research focuses on adverse childhood experiences.^20^ That literature generally concerns stressors within the family, such as abuse, neglect, or witnessing parental violence, but not usually neighborhood-level adversities. The positive associations of adverse childhood experiences with depression coupled with the known deleterious effects of chronic stress on the developing brain^21,22^ lead us to hypothesize that indirect neighborhood-level violence might have particularly harmful effects for children. Prior studies^23,24,25^ on children in war zones have generally focused on direct exposure, child soldiers, or mediators or moderators of the effects of war on children.

Most research on neighborhood-level violence and depression has been conducted in middle-income and high-income countries. Given the heterogeneity of MDD and violence exposures globally, investigating local community context across countries is a high priority. For example, the 12-month prevalence of MDD was 5.5% in the 10 high-income countries of the World Mental Health Surveys,^26^ compared with 2.7% in Nepal,^27^ one of the lowest income countries in the world and the focus for the current study.

The Chitwan Valley Family Study (CVFS)^27,28^ is a large-scale multiple cohort panel study conducted in Nepal that follows all neighborhood participants before, during, and after Nepal’s 6-year medium-intensity armed conflict between the Communist Party (Maoist) and the government security forces that cost 17 000 lives, including innocent civilians, and ended in a comprehensive peace agreement in 2006.^29^ Because this armed conflict was staged mainly using guerrilla tactics, fighting broke out in specific locations with little warning, and civilians were often unintentionally engaged. Both warring parties intentionally used several violent acts against civilians, including beatings, a tactic explicitly used to create compliance.^29^

First, we hypothesized that individuals living in neighborhoods with a higher level of beatings nearby would have a greater likelihood of developing depression than those living in neighborhoods with fewer or no beatings nearby, after controlling for individual exposure to beatings and other relevant individual and neighborhood characteristics. Second, given the unique developmental time period of childhood, we explored the association of neighborhood beatings within critical age periods at the start of the conflict. Finally, although randomized experimental designs are important for attributing cause, these are not possible (both for ethical and practical reasons) for understanding the impact of neighborhood-level violence on MDD. Therefore, we performed a novel, multivariable, multilevel matching analytic strategy^30,31^ to reduce sensitivity to unmeasured bias and more closely approximate an experimental design of our primary hypotheses.

## Methods

The cohort study was approved by the University of Michigan institutional review board and the Nepal Health Research Council. All participants provided written or verbal informed consent. This study followed the Strengthening the Reporting of Observational Studies in Epidemiology (STROBE) reporting guideline.^32^

### Study Design and Population

The CVFS, launched in 1995 with a representative sample of Western Chitwan, Nepal, is a rigorous multiple cohort panel study with low attrition.^27,33^ At launch, 151 neighborhoods were selected with probability proportionate to size, using random selection within geographical strata. All individuals aged 15 to 59 years and physically able are interviewed and followed up. All household members are tracked and become eligible for interviews at age 15 years; the sample is refreshed periodically to represent in-migrants. Data in this report include violent events and neighborhood characteristics during the armed conflict (2000-2006) and lifetime reports of MDD onset gathered from 2016 to 2018 among 10 623 participants aged 15 to 59 years (response rate, 93%). Further details about the CVFS sample and methods are available elsewhere.^27,28,34^

### Individual-Level Measures

#### Major Depressive Disorder

MDD was assessed with the Nepal-specific World Mental Health–Composite International Diagnostic Instrument 3.0,^35^ a structured diagnostic instrument administered by lay interviewers using computer-assisted methods.^35^ This instrument was paired with a life history calendar that used existing measures of individual and community events to improve recall of the timing of onset of disorders. This method has demonstrated a significantly improved measurement of lifetime experience with mental disorder over a non–life history calendar method.^34^ For the hazard of MDD, only person-years after the beginning of the armed conflict (2000) were analyzed, eliminating respondents with preconflict MDD. The remaining respondents were coded with a 1 in the year they first met diagnostic criteria according to the *Diagnostic and Statistical Manual of Mental Disorders *(Fourth Edition) for MDD and with a 0 in all other years.

#### Sociodemographic Characteristics

We included age (years), gender, ethnicity, and educational level as relevant covariates. Ethnicity in Nepal has been shown to be associated with exposures and mental health outcomes.^27,29,36^ Ethnicity was reported by participants and categorized as Brahmin-Chhetri (high-caste Hindus), Dalit (low-caste Hindus), Hill Janajati, Newar, and Terai Janajati. This categorization has been shown to capture sufficient variation in ethnicity in the study area.^36^ Educational level at interview was dichotomized into those who passed the School Leaving Certificate, a nationally standardized examination offered after the successful completion of 10th grade (coded as 1) and those who did not (coded as 0).

#### Individual Beatings

To measure whether participants had personally experienced a beating, we used the question from the traumatic events list of the Posttraumatic Stress Disorder section of the World Mental Health–Composite International Diagnostic Instrument 3.0 that asked whether the respondent had ever been beaten by anyone else other than a caretaker or romantic partner. We did not include intrafamiliar violence because the objective is to evaluate war-related violence. We created a time-varying dichotomous measure of whether a respondent was beaten.

### Neighborhood-Level Measures

#### Neighborhood Violence

The CVFS collected neighborhood-level measures of violent events from 2000 to 2006 through direct observation, focus group reports, data from the South Asia Terrorism Portal, and other news sources.^37^ Beatings within the context of the armed conflict were defined as violent acts of taking an individual or a group of individuals in control and physically hitting, slamming, stirring, or thrashing repeatedly by using hand, foot, stick, hard wood, or weapons. Each event was measured using Global Positioning System locations precise to within 30 m. We used detailed measures of timing of events to create time-varying covariates for the numbers of beatings within 1 km in each year throughout the armed conflict. Because the distribution of beatings nearby had few neighborhoods with more than 2, we classified neighborhoods as having 0, 1, or 2 or more beatings in each year.

#### Neighborhood School and Health Services

The CVFS used innovative neighborhood history calendar methods to measure respondents’ access to the nearest school and health service in walking time.^36,38,39^ We coded by whether (coded as 1) or not (coded as 0) a neighborhood had access to a school or health service within a 5-minute walk, then constructed a categorical indicator of whether a neighborhood had neither service, either service, or both services within a 5-minute walk.

### Statistical Analysis

To address the primary research questions, we conducted multilevel, discrete-time survival analysis with person-years as the unit of analysis. Logistic regression models estimated associations of individual-level and community-level exposures with MDD incidence (survival coefficients are presented as odds ratios [ORs]). We used standard multilevel hazard modeling techniques that adjust for individuals clustered within neighborhoods.^40^ To estimate potential association modification by age, stratified models of those younger than 11 years were compared with those aged 11 years and older at the beginning of the conflict in 2000. All significance tests were evaluated at *P* < .01 with 2-sided tests. Observations with missing data (1 individual) were excluded. We conducted sensitivity analyses to determine which distance thresholds to use for beatings nearby. Data analysis was performed from May 2019 to July 2020.

To simulate an experimental design, we used optimal multilevel matching methods created by Zubizarreta and colleagues.^41,42^ The treatment-control matching process tested for the presence of a neighborhood-level treatment effect of 2 or more cumulative beatings within 1 km. Such matching methods are applied to observational data to approximate the results of a true randomized experiment. The process uses the full 10 623 sample to find 197 children younger than 11 years in 2000 without exposure to 2 or more beatings within 1 km who match the 197 children who did have exposure to 2 or more beatings within 1 km at any time during the conflict. First, we used a form of cardinality matching (following the method outlined by Zubizarreta et al^41^), which simultaneously mean-balances and/or exact-matches on key covariates (on both the neighborhood and individual level) in their original form, without needing to estimate propensity scores or any other summary measure of the covariates.^42^ This process resulted in 2 matched samples of individuals who were marginally mean-balanced within 0.05 SD on 2 neighborhood-level dimensions (indicators of whether a school or health service was located within a 5-minute walk) and 3 individual-level dimensions (gender, age group [<11 vs ≥11 years], and education). Second, we exact-matched on a binary version of ethnicity (Brahmin-Chhetri and Newar [49.4% of the sample] or not) because of the key role this ethnic divide played in the armed conflict within Chitwan. Finally, we used a 2-sample *z*-test to test the association between the treatment condition (≥2 beatings within 1 km of the individual’s neighborhood during the 2000-2006 conflict period) and postconflict MDD incidence between 2007 and 2016 to 2018. Different from the hazard models, the matching analysis does not include exposure to MDD risk during the conflict period.

## Results

### Sample Characteristics

A total of 10 623 participants (5745 female [54.08%]; 4074 [38.35%] aged <11 years at the conflict start) contributed 171 899 person-years at risk of MDD during and after the armed conflict. Table 1 shows the individual descriptive characteristics of the sample. Brahmin-Chhetri was the largest ethnic group (4634 individuals [43.62%]). Fewer than one-half of participants (4067 individuals [38.28%]) completed 10th grade and passed the School Leaving Certificate examination. The lifetime prevalence of MDD at any point up until 2016 to 2018 was 15.09% of the total sample (1603 individuals), but was higher (1209 individuals [18.46%]) for those aged 11 years and older at the start of the conflict compared with those younger than 11 years in 2000 (394 individuals [9.67%]). Five hundred twenty individuals (4.90%) lived within a community where 2 or more beatings had occurred. The associations of sociodemographic characteristics with MDD are described elsewhere.^27^

**Table 1.** Descriptive Sample Characteristics of Individuals in the Chitwan Valley Family Study by Age Group

Characteristic	Participants, No. (%)		
	All ages (N = 10 623)	Aged <11 y in 2000 (n = 4074)	Aged ≥11 y in 2000 (n = 6549)
Lifetime prevalence of major depressive disorder	1603 (15.09)	394 (9.67)	1209 (18.46)
Exposure of interest: living in a neighborhood with beatings within 1 km			
0 Beatings	7788 (73.31)	2970 (72.90)	4818 (73.57)
1 Beating	2315 (21.79)	907 (22.26)	1408 (21.50)
≥2 Beatings	520 (4.90)	197 (4.84)	323 (4.93)
Covariates			
Female	5745 (54.08)	2320 (56.95)	3425 (52.30)
Ethnicity			
Brahmin-Chhetri	4634 (43.62)	1658 (40.70)	2976 (45.44)
Hill Janajati	2106 (19.82)	824 (20.23)	1282 (19.58)
Dalit	1301 (12.25)	547 (13.43)	754 (11.51)
Newar	640 (6.02)	228 (5.60)	412 (6.29)
Terai Janajati	1942 (18.28)	817 (20.05)	1125 (17.18)
Education level: passed the School Leaving Certificate examination	4067 (38.28)	2301 (56.48)	1766 (26.97)
Individual was beaten	244 (2.30)	108 (2.65)	136 (2.08)
Lived in a neighborhood with school and health services within a 5 min walk			
No nearby school or health service	4866 (45.81)	1893 (46.47)	2973 (45.40)
Either a nearby school or health service	3538 (33.31)	1353 (33.21)	2185 (33.36)
Both a nearby school and health service	2219 (20.89)	828 (20.32)	1391 (21.24)

### Neighborhood Characteristics

The study included 151 neighborhoods with a mean (SD) size of 91.11 (40.43) individuals in each. Almost one-half of individuals (4866 individuals [45.81%]) lived in neighborhoods with no school or health services within a 5-minute walk. One beating occurred within 1 km of 27 neighborhoods (17.9%), and 2 or more beatings occurred within 1 km of 15 neighborhoods (9.9%).

### Odds of MDD by Exposures of Interest

Table 2 shows 4 different discrete time survival models: the first for the total sample, the second for those younger than 11 years at the start of the conflict in 2000, the third for those aged 11 years and older at the start of the conflict, and the fourth for the total sample with the interaction of age group and neighborhood beatings. In the total sample (model 1), neighborhood beatings were not associated with developing MDD. However, children living in communities with 2 or more beatings nearby had a higher likelihood of developing MDD than those who lived in a community with no beatings (model 2, OR, 1.82; 95% CI, 1.17-2.84; *P* = .008), whereas those aged 11 years and older did not (model 3, OR, 1.02; 95% CI, 0.62-1.66; *P* = .94). There was a significant interaction between age group and neighborhood beatings (model 4, OR, 1.85; 95% CI, 1.27-2.70; *P* = .001). Only 1 beating nearby was not associated with significantly higher odds of MDD compared with no beatings in any model. Having personally experienced a beating during the conflict period, in adjusted models, was not significantly associated with respondents’ likelihood of developing MDD. Fully unadjusted estimates for the association between nearby beatings and MDD incidence are shown in eTable 1 in the Supplement. Models using different distance thresholds for nearby beatings (eTable 2 and eTable 3 in the Supplement) show that the magnitude of the association declines as distance increases. Models dividing the young age group (eTable 4 in the Supplement) show increased odds for children aged 8 to 11 years and those younger than 8 years, but not for the older children, with large confidence intervals attributable to low cell counts.

**Table 2.** Multilevel Discrete Time Hazard of Developing Major Depressive Disorder From 2000 to 2016-2018 Among Chitwan Valley Family Study Participants

	OR (95% CI)^a^			
	Model 1 (all ages)	Model 2 (aged <11 y in 2000)	Model 3 (aged ≥11 y in 2000)	Model 4 (model 1 with interaction)
Neighborhood beating events within 1 km between 2000 and 2006				
≥2 Beatings	1.34 (0.83-2.16)	1.82 (1.17-2.84)^a^	1.02 (0.62-1.66)	1.02 (0.63-1.65)
1 Beating	1.06 (0.89-1.26)	1.07 (0.85-1.36)	0.98 (0.81-1.18)	0.97 (0.80-1.17)
0 Beatings	1 [Reference]	1 [Reference]	1 [Reference]	1 [Reference]
Female	3.07 (2.64-3.56)^a^	1.78 (1.42-2.24)^a^	3.91 (3.28-4.65)^a^	3.01 (2.59-3.50)^a^
Ethnicity				
Brahmin-Chhetri	1 [Reference]	1 [Reference]	1 [Reference]	1 [Reference]
Hill Janajati	1.19 (0.99-1.43)	1.07 (0.80-1.42)	1.19 (0.97-1.46)	1.16 (0.96-1.39)
Dalit	1.60 (1.29-1.97)^a^	1.45 (1.10-1.92)^a^	1.55 (1.17-2.06)^a^	1.53 (1.25-1.89)^a^
Newar	0.76 (0.54-1.09)	0.67 (0.39-1.14)	0.81 (0.56-1.17)	0.77 (0.54-1.09)
Terai Janajati	0.93 (0.77- 1.13)	0.92 (0.70-1.20)	0.88 (0.69-1.12)	0.90 (0.74-1.10)
Education level: passed the School Leaving Certificate examination	0.68 (0.59-0.79)^a^	0.62 (0.51-0.76)^a^	0.58 (0.47-0.72)^a^	0.61 (0.53-0.71)^a^
Age	1.19 (1.16-1.22)^a^	2.06 (1.77-2.41)^a^	1.12 (1.07-1.17)^a^	1.27 (1.23-1.30)^a^
Age squared^b^	0.997 (0.997-0.998)^a^	0.983 (0.979-0.988)^a^	0.998 (0.998-0.999)^a^	0.996 (0.996-0.997)^a^
Individual beaten	1.52 (0.93-2.48)	1.68 (0.89-3.20)	1.21 (0.65-2.26)	1.45 (0.89-2.36)
Child <11 y old in 2000	NA	NA	NA	1.99 (1.62-2.43)^a^
Age group by neighborhood beatings interaction				
Child and 0 beatings within 1 km	1 [Reference]	1 [Reference]	1 [Reference]	1 [Reference]
Child and 1 beating within 1 km	NA	NA	NA	1.13 (0.88-1.46)
Child and ≥2 beatings within 1 km	NA	NA	NA	1.85 (1.27-2.70)^a^
Nearby schools and health services	0.97 (0.88-1.07)	0.91 (0.80-1.04)	1.01 (0.90-1.12)	0.97 (0.88-1.07)
Person-years, No.	171 899	70 547	101 352	171 899
Individuals, No.	10 166^c^	4074	6092	10 166

Abbreviation: NA, not applicable.

^a^ Denotes statistical significance at *P* < .01.

^b^ Age squared is a time-varying indicator of the decaying effect of age.

^c^ Some respondents are censored from these hazard models because they experienced major depressive disorder before 2000, the beginning of exposures to neighborhood violence.

The Figure shows the probability of developing MDD each year among those who were children at the beginning of the conflict living in neighborhoods with 0, 1, or 2 or more beatings nearby. Although the annual probability varied dynamically each year, the probability for those experiencing 2 or more beatings nearby was consistently higher than the probability for others.

**Figure.**
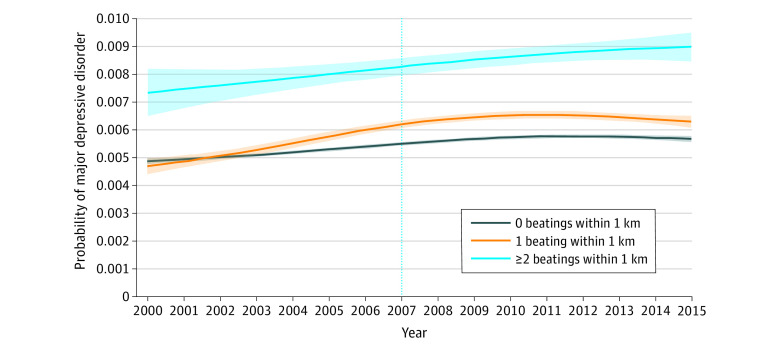
Estimated Probability of Major Depressive Disorder Among Children Younger Than 11 Years in 2000 The vertical line marks the year 2007, which represents the first year after the armed conflict ended in 2006. Lines denote means of estimated probabilities, and shaded areas denote 95% CIs.

The multivariable multilevel matching results are consistent with the discrete time survival models (Table 3). There were 197 participants who were children at the start of the conflict and living in neighborhoods that had 2 or more beatings nearby. The mean (SE) incidence of MDD among them was 12.69% (2.37%) (25 individuals) compared with 5.08% (1.56%) (10 individuals) among the matched unexposed sample (*z* = −2.66; *P* = .008). Statistically significant evidence of a treatment effect was not present in the older age group of 323 individuals. In this age group, the exposed group had an MDD incidence of 7.74% (25 individuals), compared with 6.81% (22 individuals) in the matched unexposed group (*z* = −0.454; *P* = .65).

**Table 3.** Results of Multivariable Multilevel Matching for Neighborhood Differences in Postconflict Major Depressive Disorder Incidence (Between 2007 and 2016-2018) Among Chitwan Valley Family Study Participants

Variable	Major depressive disorder incidence			
	Unmatched		Matched 1:1^a^	
	Participants, No./total (%) [SE]	*P* value^b^	Participants, No./total (%) [SE]	*P* value^b^
Aged <11 y in 2000				
<2 Beatings within 1 km	336/3877 (8.67) [0.45]	.05	10/197 (5.08) [1.56]	.008
≥2 Beatings within 1 km	25/197 (12.69) [2.37]		25/197 (12.69) [2.37]	
Aged ≥11 y in 2000				
<2 Beatings within 1 km	426/6226 (6.84) [0.32]	.54	22/323 (6.81) [1.40]	.65
≥2 Beatings within 1 km	25/323 (7.74) [1.49]		25/323 (7.74) [1.49]	

^a^ One-to one matching was performed as multidimensional mean balancing on both an individual level (binary gender, binary School Leaving Certificate passed, binary age group [aged ≥11 years cohort only]) and a neighborhood level (school on foot 5 minutes, and health service on foot 5 minutes). Exact matching was performed for binary ethnicity (Brahmin-Chhetri and Newar vs all others).

^b^ All *P* values are 2-sided.

## Discussion

This study found that living in a neighborhood with 2 or more beatings nearby as a young child during an armed conflict was associated with subsequent development of MDD, even after controlling for individual and neighborhood characteristics. This is consistent with both the life course theory that early childhood adverse events can shape the lives of individuals for decades^43^ and the literature regarding the lifelong impact of adverse childhood experiences.^20,44^ Early childhood is a sensitive period for brain development,^22^ and the impact of neighborhood-level violence can be significant, independently of personal experience.^5^ Our findings were replicated using multivariate multilevel matching to reduce unmeasured bias and approximate a treatment vs no-treatment condition design. Note that living in a neighborhood with only 1 beating nearby was not associated with likelihood of MDD in either approach, suggesting that MDD is not shaped by a single acute event, but rather multiple events.

In this setting, those who were adults when the armed conflict began appear to be more resilient to MDD, whereas those who were young children were at the highest risk. Although other studies have found neighborhood-level violence to be associated with depressive symptoms or psychological distress in adults,^7,45,46^ none, to our knowledge, have had such a long follow-up period. Effects on adults may be more short-lived or proximal than effects on children. It is possible that this age difference in risks of psychiatric disorder is present for other forms of violence nearby and other psychiatric disorders. More research is needed to understand these relationships. Nevertheless, these findings are exceptionally valuable for population-scale targeting of interventions to reduce the risks of psychiatric disorders.

It would be valuable to extend this type of research to other disorders. Externalizing disorders may be particularly important to prioritize. To the extent that externalizing disorders may contribute to violent behaviors, identification of violent exposures that increase the risk of externalizing disorders may support population-scale estimation of cross-generation and long-term perpetuation of violence. Finally, there is also a need to integrate the study of community-level exposure to violence with research on violence within the household. Ample literature^47,48^ indicates the important psychiatric consequences of violence within the household, especially for children, but there remains much to learn about the potential interactions across the multiple forms of violence; exposure to multiple forms is likely to produce more severe consequences.^49^

### Limitations

Although this study has methodological strengths, such as the large population sample nested within neighborhoods, a high response rate (93%), geolocated measures of violence during an armed conflict, a follow-up period of almost 2 decades, and an analytical approach to simulate an experimental design, there are also limitations. First, because of retrospective assessment, the incidence of MDD is likely to be underreported and respondents’ reported ages of onset may be biased.^50^ We attempted to reduce this bias by using the life history calendar to improve retrospective reporting of MDD and the timing of onset.^34^ Second, this examination of armed conflict violence focuses on only 1 type of violence, beatings. Beatings are likely to produce consequences for those living nearby because they were used explicitly to threaten nearby people during the conflict, but other forms of violence may also have important consequences. Also, because only individuals up to age 59 years were included, we could not evaluate associations for even older age groups. It is possible that the very oldest individuals are as at risk as the very youngest individuals, because prior research^51,52^ has shown the negative outcomes of neighborhood violence on elderly individuals. Furthermore, caution should be exercised because this is one finding that needs to be replicated in further studies.

## Conclusions

Although we can never randomly assign violence to communities, this large multilevel prospective panel provides evidence suggesting that the youngest members of a community are the most at risk, with mental health consequences such as MDD that last long after the neighborhood trauma. Rarely do any populations have enough resources to treat all exposed to violence. This evidence means that population-level interventions prioritizing those who were youngest at the time of the conflict may have the greatest benefits for population mental health.

## Notes

Supplement. **eTable 1.** Unadjusted Estimates of the Association Between Nearby Beatings and the Discrete Time Hazard of Developing MDD **eTable 2.** Multilevel Discrete Time Hazard of Developing MDD Among CVFS Participants Under 11 Years Old in 2000, Testing Different Distance Thresholds for Nearby Beatings **eTable 3.** Multilevel Discrete Time Hazard of Developing MDD Among CVFS Participants Aged 11 Years or Older in 2000, Testing Different Distance Thresholds for Nearby Beatings **eTable 4.** Multilevel Discrete Time Hazard of Developing MDD Among CVFS Participants in Age Groups Under 11, Under 8, and 8 to 11 in 2000
