# Supplementary material for: Exposure to Armed Conflict in Childhood vs Older Ages and Subsequent Onset of Major Depressive Disorder
Source: JAMA Netw Open. 2020 Nov 13;3(11):e2019848. doi: 10.1001/jamanetworkopen.2020.19848 (PMC7666425; doi:10.1001/jamanetworkopen.2020.19848)
Supplement: Supplement. — eTable 1. Unadjusted Estimates of the Association Between Nearby Beatings and the Discrete Time Hazard of Developing MDD eTable 2. Multilevel Discrete Time Hazard of Developing MDD Among CVFS Participants Under 11 Years Old in 2000, Testing Different Distance Thresholds for Nearby Beatings eTable 3. Multilevel Discrete Time Hazard of Developing MDD Among CVFS Participants Aged 11 Years or Older in 2000, Testing Different Distance Thresholds for Nearby Beatings eTable 4. Multilevel Discrete Time Hazard of Developing MDD Among CVFS Participants in Age Groups Under 11, Under 8, and 8 to 11 in 2000 [file jamanetwopen-e2019848-s001.pdf]

Supplemental Online Content

Benjet C, Axinn WG, Hermosilla S, et al. Exposure to armed conflict in childhood vs older ages and subsequent onset of major depressive disorder. *JAMA Netw Open*. 2020;3(11):e2019848. doi:10.1001/jamanetworkopen.2020.19848

eTable 1. Unadjusted Estimates of the Association Between Nearby Beatings and the Discrete Time Hazard of Developing MDD

eTable 2. Multilevel Discrete Time Hazard of Developing MDD Among CVFS Participants Under 11 Years Old in 2000, Testing Different Distance Thresholds for Nearby Beatings

eTable 3. Multilevel Discrete Time Hazard of Developing MDD Among CVFS Participants Aged 11 Years or Older in 2000, Testing Different Distance Thresholds for Nearby Beatings

eTable 4. Multilevel Discrete Time Hazard of Developing MDD Among CVFS Participants in Age Groups Under 11, Under 8, and 8 to 11 in 2000

This supplemental material has been provided by the authors to give readers additional information about their work.

© 2020 Benjet C et al. *JAMA Network Open.*

eTable 1. Unadjusted Estimates of the Association Between Nearby Beatings and the Discrete Time Hazard of Developing MDD

	Model 1<br>All ages<br>OR (95% CI)[a]	Model 2<br>Under 11 in 2000<br>OR (95% CI)[a]	Model 3<br>11 and older in 2000<br>OR (95% CI)[a]
**Neighborhood beating events within 1 km between 2000 and 2006**			
2 + beatings within 1 km	1.30 (0.99 - 1.70)	**1.98** (1.33 - 2.94)	0.99 (0.68 - 1.43)
1 beating within 1 km	1.11 (0.96 - 1.29)	1.34 (1.05 - 1.70)	1.01 (0.83 - 1.22)
0 beatings within 1 km (ref.)	--	--	--
**Person-Years (N)**	171,899	70,547	101,352
**Individuals (N)**	10,166	4,074	6,092

a. OR= Odds ratio; 95% CI= 95% Confidence interval; results with a p-value <0.01 are presented in bold font.

© 2020 Benjet C et al. *JAMA Network Open.*

eTable 2. Multilevel Discrete Time Hazard of Developing MDD Among CVFS Participants Under 11 Years Old in 2000, Testing Different Distance Thresholds for Nearby Beatings

	Model 1<br>1 km<br>OR (95% CI)[a]	Model 2<br>1.5 km<br>OR (95% CI)[a]	Model 3<br>2 km<br>OR (95% CI)[a]	Model 4<br>4 km<br>OR (95% CI)[a]	Model 5<br>5 km<br>OR (95% CI)[a]
**Neighborhood beating events within a specific distance[b] between 2000 and 2006**					
2 + beatings within 1 km	**1.82** (1.17 - 2.84)	1.38 (0.85 - 2.24)	1.21 (0.81 - 1.793)	1.04 (0.78 - 1.40)	1.02 (0.74 - 1.40)
1 beating within 1 km	1.07 (0.85 - 1.36)	0.98 (0.80 - 1.20)	1.03 (0.84 - 1.27)	0.90 (0.63 - 1.26)	1.00 (0.69 - 1.45)
0 beatings within 1 km (ref.)	--	--	--	--	--
**Gender**					
Female	**1.78** (1.42 - 2.24)	**1.78** (1.42 - 2.23)	**1.78** (1.42 - 2.23)	**1.78** (1.42 - 2.23)	**1.77** (1.41 - 2.23)
**Ethnicity**					
Brahmin/Chhetri (ref.)	--	--	--	--	--
Hill Janajati	1.07 (0.80 - 1.42)	1.06 (0.80 - 1.42)	1.07 (0.80 - 1.42)	1.07 (0.79 - 1.43)	1.07 (0.80 - 1.44)
Dalit	**1.45** (1.10 - 1.92)	**1.47** (1.11 - 1.94)	1.45 (1.09 - 1.93)	1.43 (1.07 - 1.89)	1.44 (1.08 - 1.92)
Newar	0.67 (0.39 - 1.14)	0.70 (0.41 - 1.17)	0.71 (0.42 - 1.20)	0.72 (0.42 - 1.21)	0.73 (0.43 - 1.23)
Terai Janajati	0.92 (0.70 - 1.20)	0.92 (0.71 - 1.20)	0.92 (0.71 - 1.20)	0.93 (0.72 - 1.21)	0.91 (0.70 - 1.19)
**Education level**					
Passed the School Leaving Certificate	**0.62** (0.51 - 0.76)	**0.63** (0.51 - 0.77)	**0.63** (0.51 - 0.76)	**0.63** (0.51 - 0.76)	**0.63** (0.51 - 0.76)
**Age**	**2.06** (1.77 - 2.41)	**2.07** (1.78 - 2.42)	**2.07** (1.78 - 2.42)	**2.07** (1.77 - 2.41)	**2.08** (1.80 - 2.42)
**Age2[c]**	**0.983** (0.979- 0.988)	**0.983** (0.979 - 0.987)	**0.983** (0.979 - 0.987)	**0.983** (0.979 - 0.988)	**0.983** (0.979 - 0.987)
**Individual Beaten**	1.68 (0.89 - 3.20)	1.66 (0.87 - 3.19)	1.69 (0.88 - 3.24)	1.72 (0.90 - 3.30)	1.72 (0.90 - 3.30)
**Nearby Schools and Health Services**	0.91 (0.80 - 1.04)	0.93 (0.81 - 1.06)	0.93 (0.81 - 1.07)	0.93 (0.81 - 1.07)	0.94 (0.81 - 1.07)
**Person-Years (N)**	70,547	70,547	70,547	70,547	70,547
**Individuals (N)**	4,074[d]	4,074	4,074	4,074	4,074

a. OR= Odds ratio; 95% CI= 95% Confidence interval; results with a p-value <0.01 are presented in bold font.
b. The distance threshold used for the exposure of interest varies by model, as labeled in the column heading.
c. Age squared is a time-varying indicator of the decaying effect of age.
d. Some respondents are censored from these hazard models because they experienced MDD before 2000, the beginning of exposures to neighborhood violence.

© 2020 Benjet C et al. *JAMA Network Open.*

eTable 3. Multilevel Discrete Time Hazard of Developing MDD Among CVFS Participants Aged 11 Years or Older in 2000, Testing Different Distance Thresholds for Nearby Beatings

	Model 1<br>1 km<br>OR (95% CI)[a]	Model 2<br>1.5 km<br>OR (95% CI)[a]	Model 3<br>2 km<br>OR (95% CI)[a]	Model 4<br>4 km<br>OR (95% CI)[a]	Model 5<br>5 km<br>OR (95% CI)[a]
**Neighborhood beating events within a specific distance[b] between 2000 and 2006**					
2 + beatings within 1 km	1.02 (0.62 - 1.66)	1.02 (0.75 - 1.39)	1.03 (0.81 - 1.31)	1.11 (0.89 - 1.38)	1.19 (0.94 - 1.50)
1 beating within 1 km	0.98 (0.81 - 1.18)	0.93 (0.78 - 1.13)	0.98 (0.81 - 1.18)	1.03 (0.82 - 1.30)	1.09 (0.82 - 1.43)
0 beatings within 1 km (ref.)	--	--	--	--	--
**Gender**					
Female	**3.91** (3.28 - 4.65)	**3.91** (3.28 - 4.65)	**3.91** (3.28 - 4.65)	**3.89** (3.27 - 4.64)	**3.89** (3.27 - 4.63)
**Ethnicity**					
Brahmin/Chhetri (ref.)	--	--	--	--	--
Hill Janajati	1.19 (0.97 - 1.46)	1.19 (0.97 - 1.46)	1.18 (0.96 - 1.46)	1.20 (0.97 - 1.47)	1.20 (0.98 - 1.48)
Dalit	**1.55** (1.17 - 2.06)	**1.56** (1.18 - 2.06)	**1.55** (1.17 - 2.06)	**1.55** (1.17 - 2.06)	**1.55** (1.17 - 2.05)
Newar	0.81 (0.56 - 1.17)	0.81 (0.56 - 1.17)	0.81 (0.56 - 1.17)	0.81 (0.56 - 1.17)	0.81 (0.57 - 1.17)
Terai Janajati	0.88 (0.69 - 1.12)	0.88 (0.70 - 1.12)	0.88 (0.70 - 1.12)	0.90 (0.71 - 1.14)	0.91 (0.72 - 1.16)
**Education level**					
Passed the School Leaving Certificate	**0.58** (0.47 - 0.72)	**0.58** (0.48 - 0.72)	**0.58** (0.47 - 0.72)	**0.58** (0.47 - 0.71)	**0.57** (0.47 - 0.71)
**Age**	**1.12** (1.07 - 1.17)	**1.12** (1.07 - 1.17)	**1.12** (1.07 - 1.17)	**1.11** (1.06 - 1.16)	**1.11** (1.06 - 1.16)
**Age2[c]**	**0.998** (0.998- 0.999)	**0.998** (0.998 - 0.999)	**0.998** (0.998 - 0.999)	**0.998** (0.998 - 0.999)	**0.998** (0.998 - 0.999)
**Individual Beaten**	1.21 (0.65 - 2.26)	1.21 (0.65 - 2.26)	1.21 (0.65 - 2.26)	1.20 (0.64 - 2.24)	1.20 (0.64 - 2.24)
**Nearby Schools and Health Services**	1.01 (0.90 - 1.12)	1.01 (0.91 - 1.13)	1.01 (0.91 - 1.12)	1.00 (0.90 - 1.11)	1.01 (0.91 - 1.11)
**Person-Years (N)**	101,352	101,352	101,352	101,352	101,352
**Individuals (N)**	6,092[d]	6,092	6,092	6,092	6,092

a. OR= Odds ratio; 95% CI= 95% Confidence interval; results with a p-value <0.01 are presented in bold font.
b. The distance threshold used for the exposure of interest varies by model, as labeled in the column heading.
c. Age squared is a time-varying indicator of the decaying effect of age.
d. Some respondents are censored from these hazard models because they experienced MDD before 2000, the beginning of exposures to neighborhood violence.

© 2020 Benjet C et al. *JAMA Network Open.*

eTable 4. Multilevel Discrete Time Hazard of Developing MDD Among CVFS Participants in Age Groups Under 11, Under 8, and 8 to 11 in 2000

	Model 1<br>Under 11 in 2000<br>OR (95% CI)[a]	Model 2<br>Under 8 in 2000<br>OR (95% CI)[a]	Model 3<br>8 to 11 in 2000<br>OR (95% CI)[a]
**Neighborhood beating events within 1 km between 2000 and 2006**			
2 + beatings within 1 km	**1.82** (1.17 - 2.84)	1.64[b] (1.10 - 2.47)	2.23 (0.51 - 9.79)
1 beating within 1 km	1.07 (0.85 - 1.36)	1.08 (0.84 - 1.38)	1.06 (0.69 - 1.63)
0 beatings within 1 km (ref.)	--	--	--
**Gender**			
Female	**1.78** (1.42 - 2.24)	**1.90** (1.46 - 2.46)	1.63 (1.08 - 2.46)
**Ethnicity**			
Brahmin/Chhetri (ref.)	--	--	--
Hill Janajati	1.07 (0.80 - 1.42)	1.13 (0.80 - 1.62)	0.96 (0.60 - 1.54)
Dalit	**1.45** (1.10 - 1.92)	**1.75** (1.28 - 2.39)	0.91 (0.44 - 1.88)
Newar	0.67 (0.39 - 1.14)	0.77 (0.42 - 1.44)	0.50 (0.180 - 1.38)
Terai Janajati	0.92 (0.70 - 1.20)	1.07 (0.77 - 1.48)	0.64 (0.38 - 1.08)
**Education level**			
Passed the School Leaving Certificate	**0.62** (0.51 - 0.76)	**0.59** (0.46 - 0.75)	0.69 (0.46 - 1.03)
**Age**	**2.06** (1.77 - 2.41)	**2.43** (1.89 - 3.11)	**1.61** (1.21 - 2.16)
**Age2**[c]	**0.983** (0.979-0.988)	**0.978** (0.971-0.986)	0.991 (0.983-0.998)
**Individual Beaten**	1.68 (0.89 - 3.20)	2.21 (1.17 - 4.16)	0.52 (0.07 - 4.07)
**Nearby Schools and Health Services**	0.91 (0.80 - 1.04)	0.96 (0.83 - 1.12)	0.78 (0.59 - 1.03)
**Person-Years (N)**	70,547	54,116	16,431
**Individuals (N)**	4,074[d]	3,108	966

a. OR= Odds ratio; 95% CI= 95% Confidence interval; results with a p-value <0.01 are presented in bold font.
b. Result has a p-value <0.05.
c. Age squared is a time-varying indicator of the decaying effect of age.
d. Some respondents are censored from these hazard models because they experienced MDD before 2000, the beginning of exposures to neighborhood violence.

© 2020 Benjet C et al. *JAMA Network Open.*
